# All-Polymer Piezo-Composites for Scalable Energy Harvesting and Sensing Devices

**DOI:** 10.3390/molecules27238524

**Published:** 2022-12-03

**Authors:** George-Theodor Stiubianu, Adrian Bele, Alexandra Bargan, Violeta Otilia Potolinca, Mihai Asandulesa, Codrin Tugui, Vasile Tiron, Corneliu Hamciuc, Mihaela Dascalu, Maria Cazacu

**Affiliations:** 1 Department of Inorganic Polymers, “Petru Poni” Institute of Macromolecular Chemistry, Aleea Grigore Ghica Voda 41A, 700487 Iasi, Romania; 2 Department of Polyaddition and Photochemistry, “Petru Poni” Institute of Macromolecular Chemistry, Aleea Grigore Ghica Voda 41A, 700487 Iasi, Romania; 3 Department of Electroactive Polymers and Plasmochemistry, “Petru Poni” Institute of Macromolecular Chemistry, Aleea Grigore Ghica Voda 41A, 700487 Iasi, Romania; 4 Research Center on Advanced Materials and Technologies, Department of Exact and Natural Sciences, Institute of Interdisciplinary Research, Alexandru Ioan Cuza University of Iasi, Bd. Carol I nr. 11, 700506 Iasi, Romania; 5 Polycondensation and Thermostable Polymers, “Petru Poni” Institute of Macromolecular Chemistry, Aleea Grigore Ghica Voda 41A, 700487 Iasi, Romania

**Keywords:** polydimethylsiloxane, polyimide, electronic skin, hydrophobic films, piezoelectric properties, pressure sensor

## Abstract

Silicone elastomer composites with piezoelectric properties, conferred by incorporated polyimide copolymers, with pressure sensors similar to human skin and kinetic energy harvester capabilities, were developed as thin film (<100 micron thick) layered architecture. They are based on polymer materials which can be produced in industrial amounts and are scalable for large areas (m^2^). The piezoelectric properties of the tested materials were determined using a dynamic mode of piezoelectric force microscopy. These composite materials bring together polydimethylsiloxane polymers with customized poly(siloxane-imide) copolymers (2–20 wt% relative to siloxanes), with siloxane segments inserted into the structure to ensure the compatibility of the components. The morphology of the materials as free-standing films was studied by SEM and AFM, revealing separated phases for higher polyimide concentration (10, 20 wt%). The composites show dielectric behavior with a low loss (<10^−1^) and a relative permittivity superior (3–4) to pure siloxane within a 0.1–10^6^ Hz range. The composite in the form of a thin film can generate up to 750 mV under contact with a 30 g steel ball dropped from 10 cm high. This capability to convert a pressure signal into a direct current for the tested device has potential for applications in self-powered sensors and kinetic energy-harvesting applications. Furthermore, the materials preserve the known electromechanical properties of pure polysiloxane, with lateral strain actuation values of up to 6.2% at 28.9 V/μm.

## 1. Introduction

The engineering of polymer composite materials at the nanometric level has enabled the development of materials suitable for advanced applications of interest to both research and industry [1]. Materials and systems that can harvest energy from the surrounding environment and can also “sense” different stimuli—pressure, touch, temperature—have been studied in the last few decades and are critical components of many technologies that include sensors for consumer applications [2,3], adaptable robotics [4], prosthetics [5], and health monitoring [6,7]. In this context, systems that can simultaneously function as an electronic skin with sensory capabilities for specific stimuli and as an energy harvester from human kinetic motion (mW to W of power) are quite rare. To date, there are relatively few classes of materials reported with such properties, and the materials used are included in classes according to the physical phenomenon employed in its construction, such as resistive materials, with capacitive behavior, and piezoelectric and piezoresistive behavior [2,8,9].

The most prominent example of an active system with multiple functionalities is the human skin. It provides a protective barrier which prevents us from the negative effects of dehydration, toxic substances, and ultraviolet radiation. For mechanical behavior and sensing, the human skin acts as touch, vibration, compression, and stretching sensor [10]. In addition to these characteristics, an artificial electronic skin should be flexible, scalable, durable, and low-cost. Also, the materials used in the fabrication of electronic skin should be three-dimensionally compliant, covering complex areas such as elbows, knees, and shoulders. Thus, the electronic skin should be stretchable with more than 50% initial length and it should be possible to cut it in pieces of desired shape and size [11]. Skin has served as a model for electronic skin sensing devices. The last twenty years have seen great improvements in pressure sensors for electronic skins, such as being able to react with ultrahigh sensitivity to minute amounts of external pressure. What is more, the use of polymers for electronic skin allows it to be ultralight in weight and be camouflaged in any environment [12].

In order to develop the sense of touch for electronic skin, there are two general approaches employing matrix arrays of flexible conformal transducers. The first approach employs Ge/Si-nanowire-array field-effect transistors (FETs) on a polyimide substrate with a pressure-sensitive layer [13]. The second approach employs microstructured polydimethylsiloxane films for pressure-sensitive capacitors as gate dielectrics of an organic FET [14]. These approaches have led to pressure sensors sensitive to pressures within 0.5–20 kPa, with response times on the order of milliseconds. The cells in the skin are self-powered; therefore, an electronic skin self-powered would emulate the natural skin. So far, multiple mechanisms were employed for self-powered electronic skins, such as piezoresistive [7], capacitive [15], piezoelectric [16], and triboelectric [17] effects. Such self-powered sensing devices can function as electronic skin Fitbits that measure multiple body parameters.

One class of materials intensively studied for multiple areas of use are piezoelectric ones. Piezoelectric devices based on traditional ceramic materials are rigid, and therefore are not flexible enough for preparing a conformable electronic skin. Also, such materials are difficult to arrange with good high-spatial density on thin substrates [18]. Contrariwise, polymer-based piezoelectric materials are attractive due to their low weight, mechanical flexibility, ease of scalability and formability, and low cost.

Of greater interest are piezoelectric stretchable polymer materials, as these are light, scalable, compliant, and low-cost. Piezoelectricity can also be observed in natural polymers [19,20,21,22]. Among different categories of piezoelectric polymers, the most studied are solid bulk polymers, where the molecular structure determines the piezoelectric properties [23]. Another category contains piezoelectric polymer composites, with integrated piezoelectric materials, mostly ceramics. Amorphous piezoelectric polymers are much less well researched than crystalline ones because they do not present responses at levels of commercial interest. The most studied are those containing the nitrile group, such as polyacrylonitrile, polyvinylidene cyanide, polyphenylethernitrile, or poly(1-bicyclobutanecarbonitrile). Polyimides with polar groups that have been investigated as piezoelectric sensors at high temperature are also of interest [24]. The stretchable piezoelectric materials enable the use of mass production methods for electronic skin that can mimic animal skin motions such as wrinkling and eyebrow movement.

In this paper, we introduce and characterize an all-polymer piezoelectric composite material type, using poly(siloxane-imide) statistical copolymers in elastomer composite films prepared with a matrix of long chain polysiloxane polymers. The materials are part of the category of bulk amorphous piezoelectric polymers [25]. The elastomer films demonstrate excellent functionality as light and flexible piezoelectric materials for potential application in low intensity kinetic energy harvesting (such as from human walking) and for pressure sensors, as well as barrier layer with low water vapor sorption that protects against moisture and humidity (<0.8% water vapor sorption relative to sample dry weight), thus fulfilling the material requirements for an artificial electronic skin.

## 2. Results and Discussion

### 2.1. Synthesis of Poly(siloxane-imide) Copolymers

**Scheme 1:**
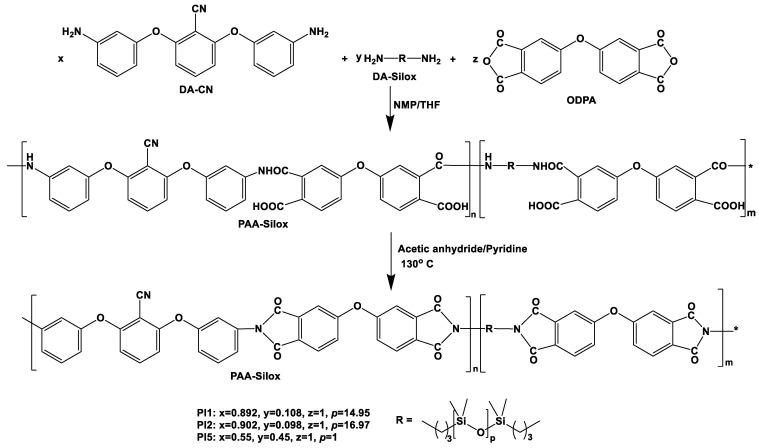
Preparation of poly(siloxane-imide) copolymers containing CN groups, PIa.

Three poly(imide-co-siloxaneimide) copolymers, PI1, PI2, and PI5 (Scheme 1), whose ratios differ between the two sequences, as does the length of the siloxane segment, were prepared according to a two-step process, following a previously described procedure [26,27]. In the first step, solutions of polyamic acids, PAA-Silox1-3, were prepared from polycondensation reactions of 4,4′-oxydiphthalic anhydride (ODPA) with mixtures of two diamines, 2,6-bis(3-aminophenoxy)benzonitrile (DA-CN), and α,ω-bis(3-aminopropyl)oligodimethylsiloxane (DA-Silox1-3). In the second step PAA-Silox1-3 were converted to the corresponding imide structure PI1, PI2, and PI5 using chemical imidization. The copolymer structures were checked using FTIR spectroscopy (Figure S1) and ^1^H-NMR (Figure S2), while the molecular weights were estimated by GPC (see below Section 3.1 Materials).

### 2.2. Preparation of Elastomeric Composites

**Scheme 2:**
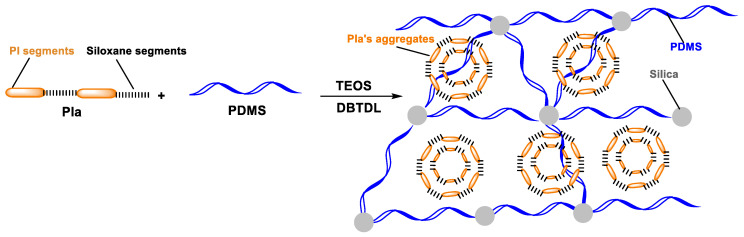
Schematic procedure for the obtaining of siloxane-based elastomers PIa-b.

The three PIa copolymers (with a = 1, 2, 5 designating each copolymer prepared), previously grounded, were incorporated in 2, 5, 10, 20 wt% within a high molecular weight polydimethylsiloxane-α,ω-diol (Mn = 370,000 g·mol^−1^) by mixing in a solution with chloroform (Table 1). The presence of di- or oligodimethylsiloxane fragments in the structure of the polyimide creates the prerequisites for their better compatibility with the silicone matrix. Self-assembly in a non-polar environment provided by the PDMS matrix could lead to aggregates with oligodimethylsiloxane fragments on the outside and the polar polyimide blocks inside the aggregates. The mixtures were processed into film and stabilized by cross-linking with TEOS in the presence of DBTDL, which was added to the mixture just before casting (Scheme 2). The elastomers prepared with PDMS have high-durability, which allows for parts with limited operational failures and full operational uptime [28,29], long running parts without decrease of performance [30,31], and can be prepared with a variety of other materials for improved electrical and mechanical properties. This array of properties was compelling for the preparation of new materials that bring together the elastomeric nature of PDMS with the expected piezoelectric properties of polyimides, which by themselves form brittle films. The obtained free-standing films were investigated from the point of view of morphology, surface properties, mechanical, dielectric, electromechanical, and piezoelectric responses.

### 2.3. Characterization of the Elastomeric Molecular Composites

#### 2.3.1. Scanning Electron Microscopy

**Figure 1:**
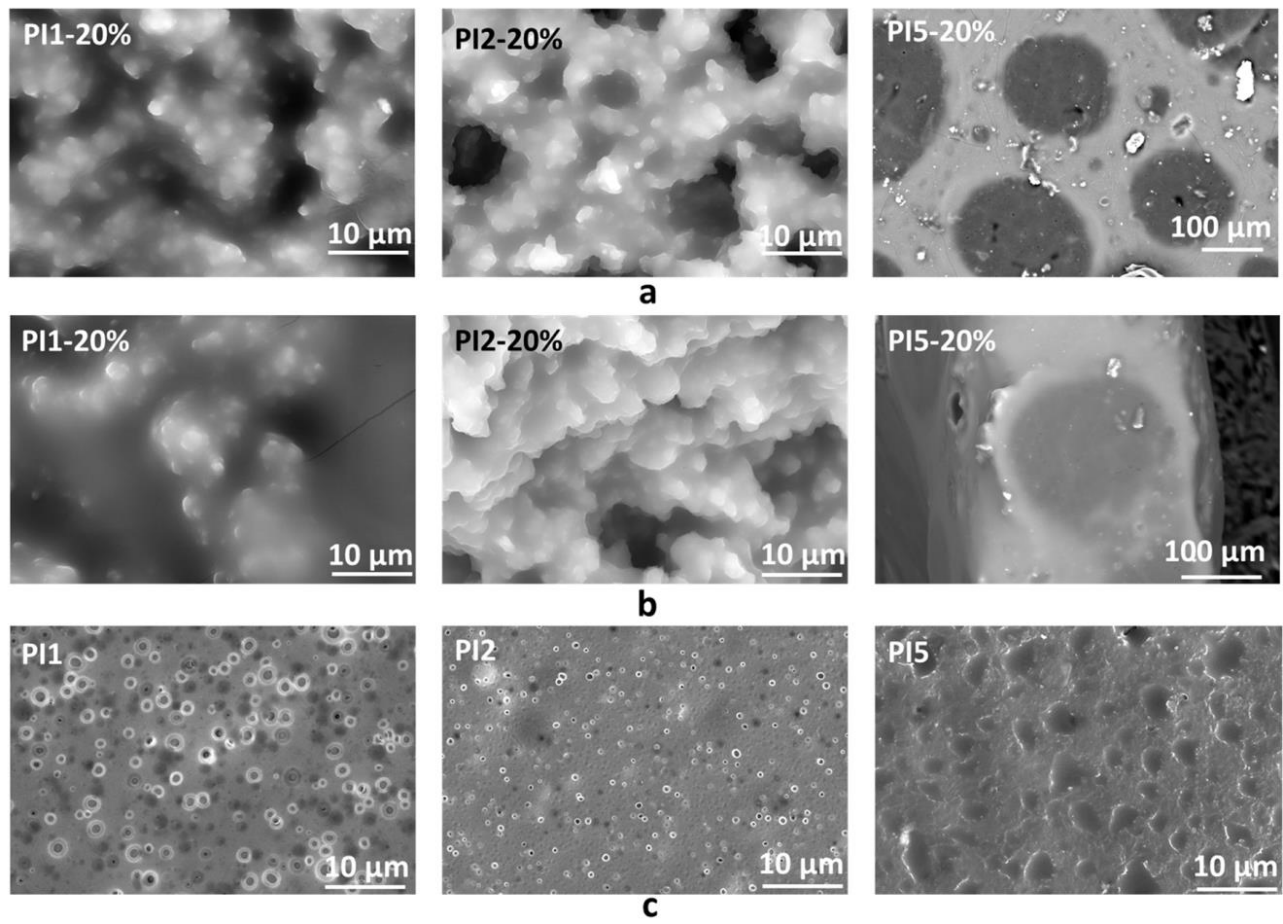
SEM images of the PIa-b siloxane-based elastomer films: (**a**)—on the surface, (**b**)—in section (recorded by using Circular Back Scattered Detector, CBS); (**c**)—film surface of the initial PIa samples (recorded by using Everhart Thornley Detector, ETD).

The surface and cross-section morphology of composite films was evaluated using SEM microscopy (Figure 1a,b and Figure S3). The images indicate the presence of micron-sized, roughly spherical aggregates of PI, and their relatively uniform distribution within the silicone matrix. This is especially noticeable in the case of the PI1 and PI2 series, which contain longer siloxane segments, where *p* is 14.95 and 16.97, respectively, which favors the self-assembly of the PI copolymer in the hydrophobic silicone matrix. In the case of PI5, where the silicone sequences, although more in number, are very short, this phenomenon is diminished.

#### 2.3.2. Dynamic Water Vapor Sorption

**Figure 2:**
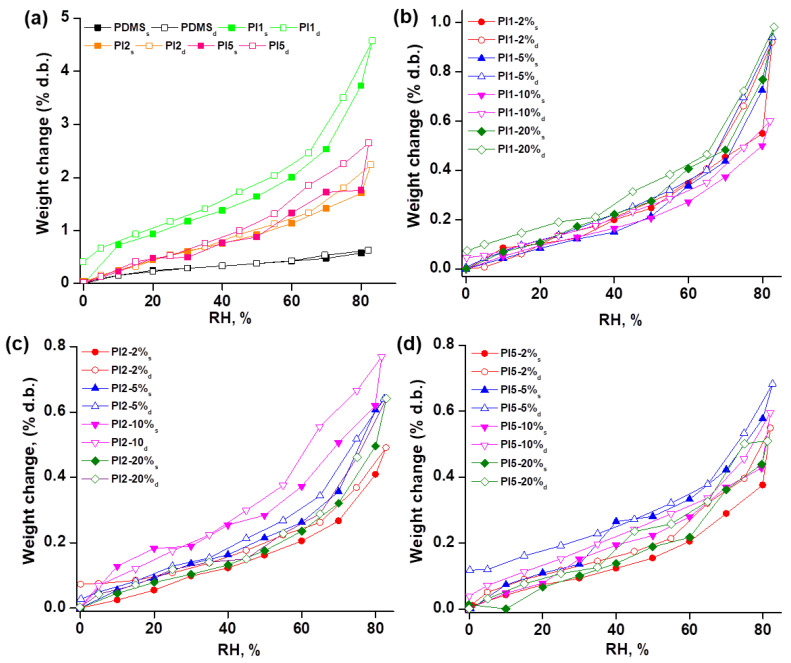
Water vapor sorption capacity for: (**a**) reference samples; (**b**) PI1; (**c**) PI2; (**d**) PI5 composite series, where *s* and *d* subscript coefficients represent the sorption and desorption isotherms.

The water vapor sorption capacity of the developed composites was tested to determine the protection ability against humidity necessary to fulfill the requirements of materials for further application, as an artificial electronic skin, for example. The water vapors sorption–desorption isotherms recorded in dynamic mode are shown in Figure 2. In the PIs samples, the existence of polar side groups and highly flexible siloxane chains interfere with the dense and well-packed polyimide structure. The PDMS reference sample, presented a water vapor sorption capacity of 0.62 wt% (Figure 2a), while the polyimide samples had the following maximum sorption values: PI1-4.58 wt%, PI2-2.24 wt%, and PI5-2.65 wt%, in accordance with the content in siloxane segments and their length. The sample PI1 shows the highest retention capacity for water vapors; the presence of the free carboxylic groups and low crosslinking density could explain this behavior [32]. The presence of the siloxane component does not inhibit the moisture sorption capacity in the samples containing polyimide [33]. This implies that the siloxane chains are excluded from the polar polyimide regions, and the local density of polar groups (imide, nitrile, hydroxyl) can be higher. As can be seen in Figure 2b, the sample PI1s retains 0.41% water in its mass after the sorption–desorption cycle, while the other samples retain negligible quantities (0.02–0.04%) (Figure 2c,d). In the three PIa-b series, the water vapor sorption capacity does not change significantly (0.92–0.98, 0.64–0.77, and 0.51–0.68 wt%, respectively). This indicates the high hydrophobicity and that the amount of water remaining in the sample after the sorption–desorption cycle is negligible in all cases. Thus, all samples show low values for water sorption, and after desorption, the samples return to their initial state. This behavior demonstrates that the morphology of the samples is not affected by water vapor sorption capacity.

#### 2.3.3. Static Contact Angle and Surface Energy

The hydrophobic character of composite materials is sustained also by static contact angles and surface energy values. As can be seen in Table 2, the incorporation of longer siloxane segments in the structure of polyimides has the effect of a slight increase in their hydrophobicity, the contact angle with water increasing from 93 degrees (as the full organic polyimide has) to 105 degrees in PI2 with the longest siloxane segments in the structure, but all of them are below 115°, which is the PDMS used as a matrix for the prepared composites. As a result, it would be expected that, by incorporating polyimides in the matrix, their hydrophobicity would decrease somewhat, which does not happen. Incorporation of statistical copolymers in small amounts up to 5% has an insignificant influence on static contact angles, work of adhesion, or interfacial tension for a solid-liquid system, especially since, as is known, the silicone component, due to its flexibility and low surface tension, always migrates to the surface. In the meantime, by increasing the amount of copolymers incorporated in PDMS matrix, the materials became more hydrophobic, taking into account the values for parameters of interest. Polymeric surface wettability plays a significant role in the design of the materials with tailored applicability, for example electronic skin. The static contact angle was measured and the results are shown in Table 2. The water contact angle value recorded for the PDMS sample was 115 degrees. All composite films are also characterized by a hydrophobic surface, with a water contact angle in the range of 112–128 degrees. The inclusion of PIa copolymer into the PDMS matrix has two opposite effects on the hydrophobicity of the polymeric surface: a low concentration (2–5 wt%) leads to a slightly lower value of the water contact angle (112–114 degrees) for samples with PI1 and PI2, and a higher concentration (10–20 wt%) increases the hydrophobicity, with the water contact angle reaching 127 degrees (PI1-20% and PI2-20%).

The increase of the contact angle is directly connected with the increase in the surface roughness of the tested film samples, which in turn is directly correlated with the increase in the content of polyimide copolymer, as can be seen in the AFM data (see below). Thus, in the composite samples, the copolymer self-assembles into micron-sized aggregates. On the outer surface of such aggregates, the siloxane segment of the copolymer is in direct contact with the PDMS matrix. The number of the aggregates formed increases with increasing content of polyimide copolymer, which creates a rough surface of the free-standing films of composite materials. In turn, the nanometer-sized roughness variations impact the contact angle of each sample of composite material.

PI5 composite films showed a different trend; the water contact angle did not exceed 116 degrees, even for high copolymer concentrations (PI5-20%). The lower values for the contact angle in this case can be assigned to a lower roughness of the films (see below), similar to that for pure silicone film.

The surface free energy (SFE) with its components, polar $\left(\gamma_{sv}^{p}\right)$ and dispersive ($\gamma_{sv}^{d}$) contributions, is very important in understanding the chemistry of surface-based phenomena, which are essential in different applications including adhesion, coating, printing, etc. Based on the Young (Equation (1)) [34], Owens and Wendt (Equation (2)) [35], and Fowkes equations (Equation (3)) [36], the SFE of the composite films and its components were calculated using two reference liquids (water and ethylene glycol) (Figure S4):

(1) $$\gamma_{sv}=\gamma_{sl}+\gamma_{lv}\cos\theta$$

(2) $$\gamma_{lv}\left(1+\cos\theta \right)=2\sqrt{\gamma_{lv}^{p}\gamma_{sv}^{p}}+2\sqrt{\gamma_{lv}^{d}\gamma_{sv}^{d}}$$

(3) $$\gamma_{sv}=\gamma_{sv}^{d}+\gamma_{sv}^{p}$$

where *θ* represents the static contact angle, *γ_sv_* represents the surface free energy of solid in equilibrium with the saturated vapor of the liquid, *γ_lv_* represents the surface free energy of liquid in equilibrium with its saturated vapor, *γ_sl_* represents interfacial free energy of solid to liquid,$\;\gamma_{sv}^{p}$, $\;\gamma_{lv}^{p}$ are the polar component of the surface free energy and $\gamma_{sv}^{d}$, $\;\gamma_{lv}^{d}$ are the dispersive component of the surface free energy.

Another parameter that can be calculated based on the contact angle value is the spreading coefficient (*S_c_*) which will dictate and predict the wettability of the polymer surface. The spreading coefficient is based on the adhesion (*W_a_*) and cohesion work (*W_c_*) [37]:

(4) $$S_{c}=W_{a}-W_{c}$$

The adhesion work which characterizes the binding between the phases was calculated based on the Young–Dupré equation [34,38]:

(5) $$W_{a}=\gamma_{lv}\left(1+cos\theta \right)$$

where $\gamma_{lv}$ represents the surface tension of the liquid and *θ* is the static contact angle.

The cohesion work represents the surface resistance to the liquid:

(6) $$W_{c}=2\gamma_{lv}$$

**Figure 3:**
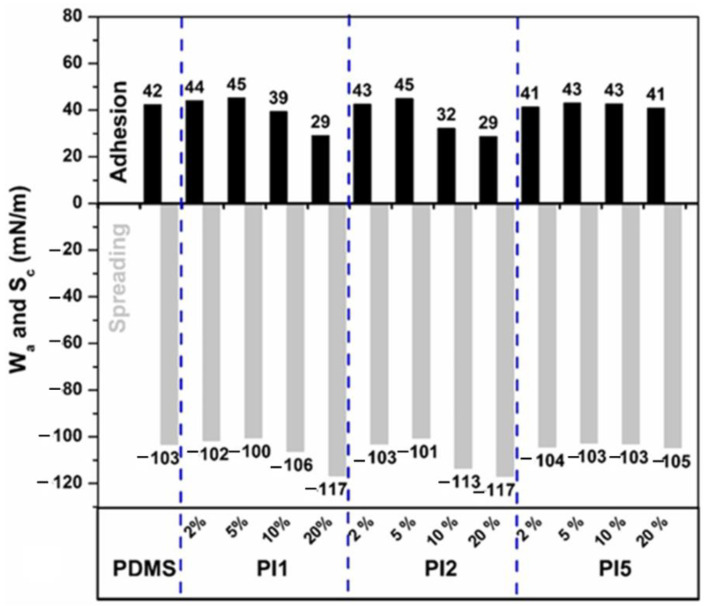
Work of adhesion and spreading coefficient of the composite films.

The calculated values for the adhesion work and spreading coefficient are illustrated in Figure 3. Based on Equation (5), the work of the adhesion is inversely proportional to the contact angle, with a lower contact angle leading to a higher adhesion work value. Low concentration of copolymer filler leads to a higher value of the *W_a_* (43–45 mN/m) for PI1 and PI2 composite. Further increasing of the filler concentration (20%) into the PDMS matrix has an opposite effect, with the decreased binding between liquid and polymer surface leading to a *W_a_* value of 29 mN/m. The values of the adhesion work are not significantly influenced by the filler concentration for PI5 samples. When *S_c_* > 0, the surface is completely wetted by the tested liquid and if *S_c_* < 0, the cohesion forces between liquid molecules are higher, and the liquid partially wets the solid surface [39,40]. All composite films are characterized by negative values indicating that the liquid did not spread across the polymer surface (Figure 3). Thus, the low values of the *S_c_* dictate the hydrophobic character of the composite film surfaces.

#### 2.3.4. Mechanical Tests

**Figure 4:**
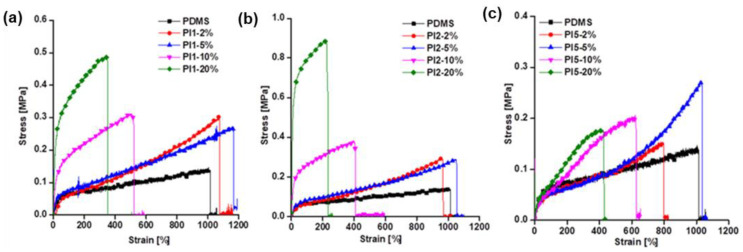
Mechanical stress-strain curves for (**a**) PI1, (**b**) PI2 and (**c**) PI5 composite series reported to reference sample, PDMS.

The mechanical properties evaluated from the elongation at break tests of the three series of composite films (PIa-b%) are presented in Figure 4, in comparison with a sample made of pure PDMS, the reference sample. All PIa-2% samples have a similar behavior with the PDMS reference; the amount of copolymer incorporated in the PDMS matrix has an insignificant influence. For all PIa-5% samples, a higher elongation at break (up to 1165%) was obtained as compared to the PDMS sample. In the meantime, for the samples with 10 and 20 wt%, the strain at break is reduced almost by half, and the Young modulus increases considerably.

The introduction of larger percentages of polyimides in the PDMS matrix leads to increased Young modulus values, this effect being most visible for the samples prepared with PI2, where the Young modulus increases from 0.22 MPa at 2 wt% PI2 content (similar with reference PDMS) to 6.6 MPa for sample PI2-20% (Table 3). A similar behavior is visible for the stress at the break (T_nm_). The use of polyimides increases the plastic component of deformation in stress-strain tests. While the samples with 2 or 5 wt% polyimide show an elastic deformation for up to 20% strain and a reduced hysteresis after the first five cycles (Figure S5), the samples with 10 and 20 wt% polyimide show a large remanent plastic deformation and a large hysteresis even after 10 cycles of stress-strain tests (Figure S5). However, the results of the mechanical stress-strain tests demonstrate that all samples behave as elastomers, with large values for the breaking strain of well over 200% strain, even for the sample with the largest value of the Young modulus (PI2-20).

#### 2.3.5. Dielectric Spectroscopy

**Figure 5:**
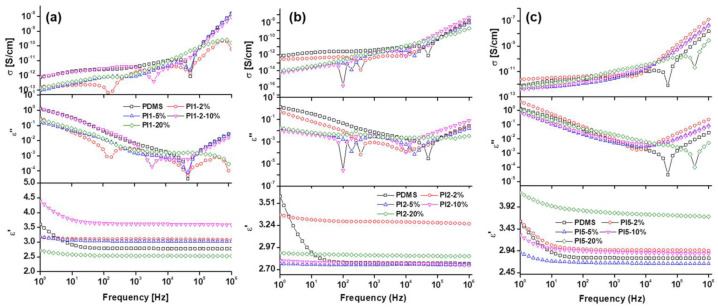
Dielectric properties of the tested films including relative permittivity (ε′), dielectric loss (ε″), and conductivity (σ) for samples prepared with PI1 (**a**), PI2 (**b**), and PI5 (**c**).

There is a notable increase in the values of dielectric constants occurring with the increase in content of polysiloxane-imide copolymers from 0 to 20 wt% (Figure 5). This is concurrent with the formation of hard domains of polyimide (Figure 1c) due to the phase separation of the siloxane-imide and imide segments from the copolymers in the composite films. In this study, each of the three copolymers tested has an average length of the soft siloxane segment and a defined length of the hard polyimide segment, with three different ratios of soft/rigid segments. The films are formed from a solution of self-assembled granular microdomains with isotropic distribution of ~1 μm confining the crystalline piezoelectric imide-based polymer. Simultaneously, the soft siloxane chain segment can adopt any conformation and has high mobility, leading to the formation of a homogeneous composite at macroscale [41]. Similar to other thermoplastic elastomers, supramolecular interactions of the hard polyimide segment can take place by π–π stacking between aromatic rings of 3,3′,4,4′-benzophenonetetracarboxylic dianhydride and by hydrogen-bonding interactions of the nitrile and carbonyl functions [42,43,44]. The dielectric properties show the influence of the phase separation, where the composites with 10 and 20 wt% of copolymer have larger dielectric constant values in comparison with the starting siloxane and polyimide copolymers, due to the confined microdomains of polyimide segments [45].

There is a relatively small influence on the values of the dielectric properties of interest (dielectric constant, dielectric loss, conductivity) when samples are subjected to humid atmosphere or even when they are tested after being immersed in distilled water for 30 min and soaking excess water with clean wipes before measurements (Figure S6). In the range of frequencies used for tests (10^0^−10^6^ Hz), the dielectric constant is mostly within the range of 2−3.5, the dielectric loss is between 10^−4^−10^0^. At low frequencies (10^0^−10^1^ Hz) the samples prepared with PI5 show a dielectric constant >6 and a dielectric loss of ε″~10^1^ for tests performed after wetting the sample in water. This behavior can be attributed to residues of water in the polyimide phase separated at the surface of the film. The conductivity of the samples occurs in the region specific to insulating materials (σ < 10^−8^ S/cm) on the entire range of frequencies tested. The stability of the properties of composite materials to moisture (Figure 2, Table 2) and to water (Figure S6) makes these suitable candidates for the development of pressure sensors and artificial electronic skin.

### 2.4. Evaluation of Some Functional Capabilities of Elastomeric Composites

#### 2.4.1. Electromechanical Actuation

**Figure 6:**
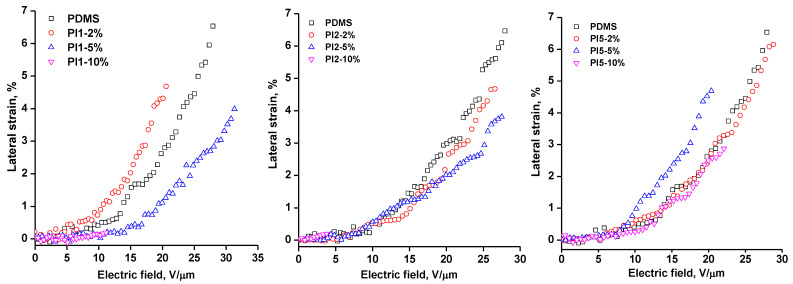
Dielectric actuation of the composite films.

Composite materials can act as electrostatic actuators with compliant electrodes deposited on the free-standing films when a direct current is applied, thanks to their dielectric nature. The capability of actuation of the composite samples was investigated in electromechanical tests using circular actuators, and the lateral actuation displacement was optically measured using a digital camera in order to evaluate the electrode extension [46]. An increasing voltage step was applied to the samples until a breakdown through the material occurred. In the series based on PI1, the largest lateral actuation strain at 20 V/μm was obtained for the material with the lowest wt% of PI1, while the highest maximum lateral actuation strain of s_max_= 4% was obtained for PI1-5% at 31 V/μm (Figure 6). The highest actuation strain at the lowest electric field was observed for PI1-2% with a s_max_= 4.7% at 20 V/μm. For PI1-10% the actuation is insignificant. Regarding the series based on PI2, the highest maximum lateral actuation strain of 4.66% was obtained for PI2-2% at 27 V/μm. In this series, the actuation strain of the tested materials were lower than the PDMS ones. In the PI5-based series, the largest lateral actuation strain at 20 V/μm was obtained for PI5-5%, while the highest maximum lateral actuation strain of 6.12% was obtained for PI5-2% at 28.9 V/μm. The highest actuation strain at the lowest electric field was observed for PI5-5% with a s_max_= 4.69% at 20 V/μm. The samples prepared with 20% polyimide (PIa-20%) showed an electric breakdown at values below 5 V/μm, thus making them unsuitable for electrostatic actuation. The variation of values for lateral actuation strain is overall correlated with the values for the breaking strain (Figure 4). The samples with the lower Young modulus and the larger values for breaking strain also show larger values for lateral strain actuation (Figure 6).

#### 2.4.2. Piezoelectric Response

Different materials, either particulate or solid polymer films, can confer piezoelectric properties onto composite materials with a siloxane matrix which is not a piezoelectric material. Polyimides have demonstrated piezoelectric properties [47] as these materials are non-symmetric, polar, and insulating. The use of such polyimides as filler materials can confer piezoelectric behavior onto composite films with silicone matrix [48,49,50].

The piezoelectric behavior of the composite films was studied with a multimode AFM setup (NT-MDT SolvePro) [51]. The local piezoelectric response was quantified using PFM imaging in one imaging session, with the same cantilever and the same laser position. When a localized electric field is applied through the conductive cantilever (with platinum conductive coating (NT-MDT), with a radius of curvature of R = 35 nm, contact resonance frequency of f = 250 kHz, and a spring constant of k = 0.2 N/m), it is possible to reduce the electrostatic contribution to the PFM signal by using a soft cantilever which has the added benefit of improving the piezoresponse’s sensitivity [47], while avoiding the damage to the soft siloxane matrix test samples specific for stiff cantilever tips. The samples were imaged by scanning an area of 20 × 20 µm^2^, in contact mode with a loading force of 2.72 nN, drive voltage of 1 V, and scan rate of 0.5 Hz, collecting PFM images of surface 2D and 3D topography, magnitude, and phase; for accuracy, dual calibration was used: first based on detector sensitivity and second with a lithium niobate test pattern using a known piezoelectric coefficient [47].

Before recording the piezoelectric response of the samples, calibration with a test sample of poled lithium niobate was used to calibrate the PFM [51], where the calibration factor was used to further convert the measured amplitude of the piezoresponse signal (*h*) to value for the effective *d*_33_, with a known amplitude of the probing AC voltage (*A*) [47]:

*h* = *γAFMd*_33_*A* (7)

**Figure 7:**
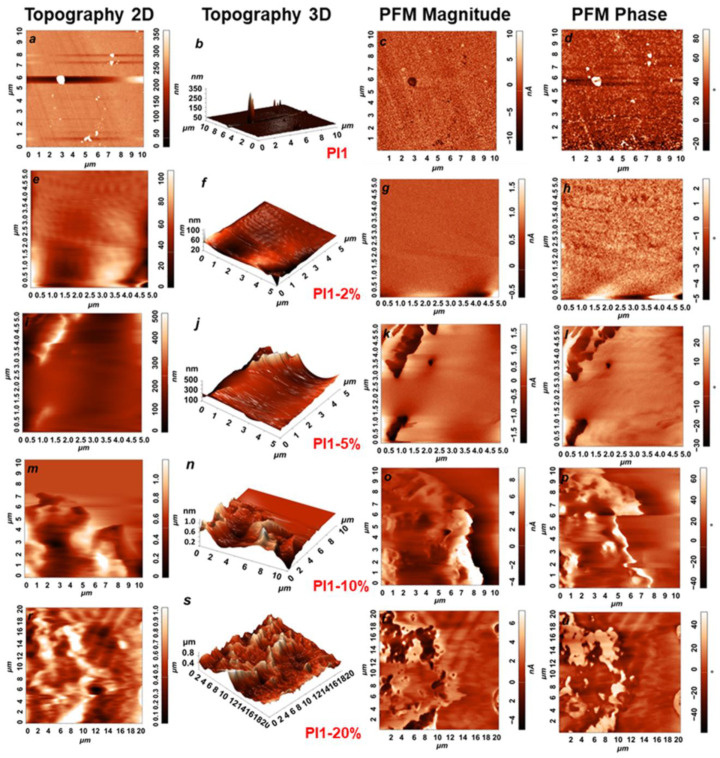
PFM images: surface 2D topography (*a*,*e*,*i*,*m*,*r*), surface 3D topography (*b*,*f*,*j*,*n*,*s*), magnitude (*c*,*g*,*k*,*o*,*t*), and phase (*d,h*,*l*,*p*,*u*) for samples prepared with PI1.

Figure 7 shows surface 2D topography (*a*,*e*,*i*,*m*,*r*), surface 3D topography (*b*,*f*,*j*,*n*,*s*), magnitude (*c*,*g*,*k*,*o*,*t*), and phase (*d*,*h*,*l*,*p*,*u*) for samples prepared with polyimide copolymer PI1. The surface area of rough domains (roughness > 10 nm) increases with the content of polyimide in the sample. Similarly, there is an increase in multiple contrasts both in magnitude and phase images with increased content of polyimide, which is specific to an increasingly widespread piezoelectric response and the different domain structures have different orientations of polarization. Each uniformly polarized region is similar with topographic features. The morphology features of the samples are significant for their influence on the piezoelectric properties, since a sample of pure silicone tested under the same PFM conditions showed no piezoelectric response.

The content of polyimide in the composite samples influences the piezoelectric response recorded: the average values of piezoelectric coefficient increase from 0.5 to 1.7 pm/V for samples with PI1, and from 0.4 to 1.5 pm/V for samples with PI5. For samples with PI2, the value of the piezoelectric coefficient does not change with the polyimide content, and it hovers around 2.5 pm/V (Table 4). These values are lower than those reported for polyimide-based piezoelectric films [52]. However, the materials are not brittle as is the case for films made from pure polyimide, and furthermore the samples are both flexible and stretchable (Figure 4 and Figure S5). Compared with samples made from pure piezoelectric materials, such as polyimides or other classic piezoelectric materials the value of 2.5 pm/V is extremely favorable, as the samples tested have a content of only 20 wt% or less of piezoelectric polyimide.

#### 2.4.3. Pressure Sensors and Energy Harvesting Data Analysis

**Figure 8:**
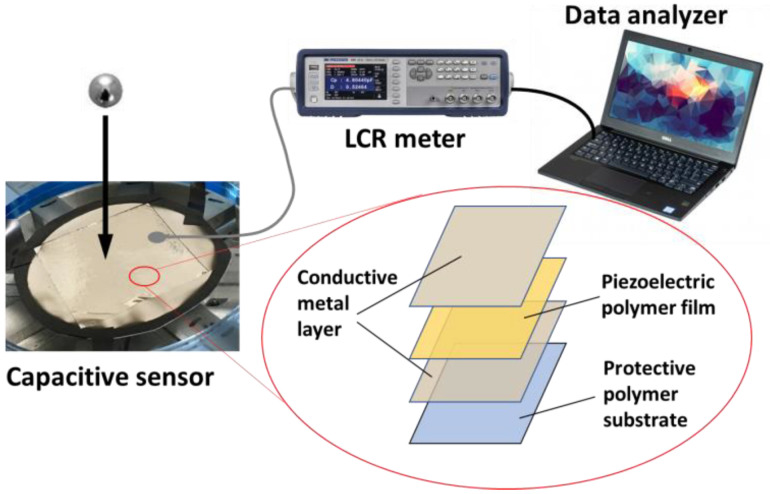
Setup for capacitive pressure sensors based on piezoelectric stretchable polymer films.

One of the main objectives of this study was to put together polysiloxane as the dielectric and polyimide as the piezoelectric component, in a new composite elastomer-type material [47]. Each sample was tested as free-standing film (thickness < 100 microns) by dropping a 30 mm diameter ball onto the sample’s surface from a height of 10 cm, as shown in Figure 8.

**Figure 9:**
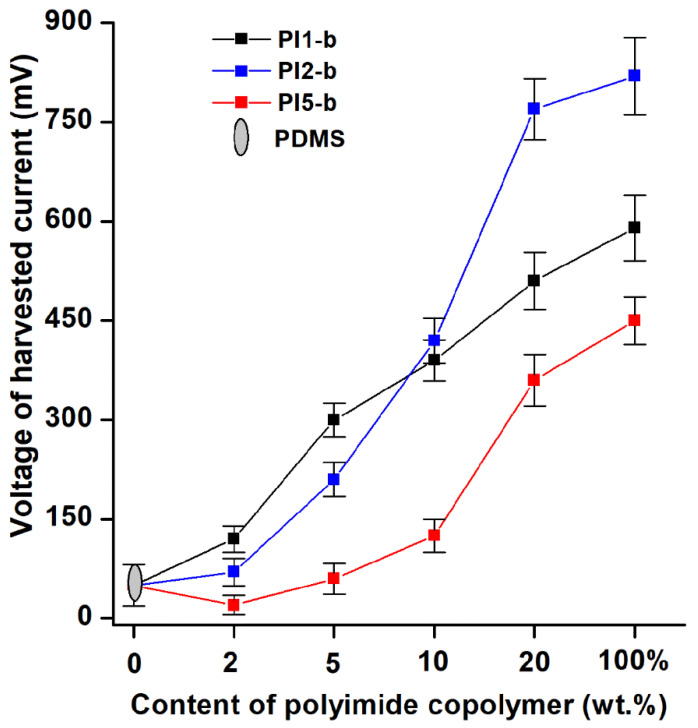
Multiresponse elastomer protective film as a piezoelectric energy harvester and pressure sensor.

The kinetic energy of the ball was directly converted by the composite piezoelectric films into an electric current, and the value of the voltage of the signal represents the amount of energy harvested from the tested sample as a direct current. The setup (Figure 8) operates under an applied direct voltage of 1 V and has a response time between 50–100 ms. The samples tested in this setup showed a response for an applied force of 0.005 N, corresponding to the films being touched with a 0.5 g weight. The maximum value of the harvested voltage is approximately 600 mV for samples of pure PI1, 800 mV for samples of pure PI2, and 450 mV for samples of pure PI5, 100% being films made from pure PI1, PI2, and PI5, respectively (Figure 9). The value of the voltage for the harvested current is around 50 mV for samples of pure PDMS. With the addition of a low concentration (2 wt%) of polyimide copolymer, it is noticeable that the values of the harvested current are similar to pure PDMS. Overall, the values of the voltage recorded for the composite materials are lower than the voltage recorded for samples made of pure polyimide copolymer. However, increasing the concentration of polyimide copolymer in composites drives the values of the voltage signal higher (Figure 9). For samples prepared with 20 wt% polyimide copolymer (PI1-20%, PI2-20%, and PI5-20%), the values of the voltage are close to 90% of the voltage for the specific pure PIa polyimide copolymer, making the elastomer films useable for energy harvesting from walking and as a pressure sensor.

## 3. Materials and Methods

### 3.1. Materials

A polydimethylsiloxane-α,ω-diol (PDMS) with molecular weight Mn = 370,000 g·mol^−1^ and polydispersity index PDI = 1.73 was used as a matrix. The polymer was synthesized using cationic ring-opening polymerization of octamethylcyclotetrasiloxane in the presence of sulfuric acid, following the procedure previously described in [53]. Tetraethylorthosilicate (TEOS) was purchased from Merck and was used as received. Dibutyltin dilaurate (DBTDL) was supplied by Sigma–Aldrich and was used as received. For the synthesis and characterization of the poly(siloxane-imide) copolymers PI1, PI2, and PI5 see Section 3.3.

### 3.2. Methods of Characterization

The *IR spectra* were registered on Bruker Vertex 70 FT-IR equipment in transmission mode, in the 400–4000 cm^−1^ range, with a resolution of 2 cm^−1^ and 32 scans, at room temperature. The *NMR spectra* were recorded on a 400 MHz Bruker spectrometer in CDCl_3_ at room temperature. Chemical shifts are reported in δ units (ppm) and refer to the internal deuterated solvent CDCl_3_ calibrated at 7.26 ppm. GPC measurements were made in CHCl_3_ on a PL-EMD 950 chromatograph—evaporative mass detector. The calibration was performed with polystyrene standards. *SEM images* were taken using a Quanta 200 scanning electron microscope (FEI Company) by using a large-field detector (LFD) and a Verios G4 UC scanning electron microscope (Thermo Fisher Scientific) with circular back scattered (*CBS*), and Everhart Thornley (ETD) detectors, in a low vacuum mode. *The moisture sorption behavior* of the samples was studied *in a dynamic regime* at 25 °C, in the 0–90% relative humidity (RH) range, using the fully automated gravimetric analyzer IGAsorp fabricated by Hiden Analytical, Warrington (UK). The vapor pressure was increased in 10% humidity steps, with a pre-established equilibrium time between 15 and 30 min. For each step, the increase of the weight was measured by electromagnetic compensation between tare and sample when equilibrium was achieved. The cycle was ended by decreasing the vapor pressure in steps, which permitted us to obtain the desorption isotherms. The drying of the samples before sorption measurements was realized at 25 °C in flowing nitrogen (250 mL/min) until the weight of the sample reached equilibrium at RH < 1%. *Dielectric spectroscopy measurements* were carried out with a Novocontrol Concept 40 broadband dielectric spectrometer device equipped with an Alpha-A high performance frequency analyzer. The samples, as free-standing films, were placed between two plated electrodes. Measurements were performed in dry nitrogen atmosphere and the amplitude of the external electrical field was equal to 1 V. The dielectric spectra were recorded under isothermal conditions, in broad frequency (10^0^−10^6^ Hz) and temperature ranges (from −150 °C to 220 °C). The temperature was controlled with a standard Novocontrol Quatro Cryosystem device. The *mechanical stress–strain tests* were conducted on an Instron 3365 test instrument, Norwood MA, USA, at an extension rate of 20 mm min^−1^ at ambient temperature. Actuation measurements were made on circular films of 50 mm in diameter, 20% equiaxially prestrained [54] and fixed between two circular frames. Circular electrodes of carbon black powder (8 mm in diameter) were deposited concentrically on both sides of the polymeric film. The lateral actuation displacement was optically measured using a digital camera and software in order to evaluate the electrode extension [46]. *Static contact angle measurements* were carried out using the sessile drop method at room temperature on a CAM-101 contact angle system from KSV Instruments, Helsinki, Finland. The contact angle instrument was equipped with a liquid dispenser of 1 mL Hamilton syringe (Hamilton Company Reno Nevada) and a video camera. A drop of liquid (1 μL) was placed on the polymer surface and the contact angle was measured immediately. Ten photos were recorded at an interval of 0.016 s. At least five measurements were performed for each liquid to obtain the contact angle value and the average values were reported. The standard deviation between the values obtained from all performed tests was less than 4%. The *deposition of a conductive metal layer* for the signal recording was done with the technique of Al film deposition through pulsed laser ablation, PLD (pulsed laser deposition). The experimental arrangement of the PLD technique uses a classic configuration, which involves placing the target and the substrate face to face in parallel planes. The conductive materials obtained are thin metallic layers of Al deposited on a polymeric substrate (Figure 8). The experimental parameters for obtaining conductive thin films by PLD are as follows: KrF laser with a wavelength of 248 nm, pulse duration 25 ns, pulse repetition frequency 20 Hz, laser fluence 1.5, 2 J/cm^2^ for a constant target-subtracted 7 cm distance, total number of pulses 18,000, Al target, purity 99.99% (GoodFellow), deposition pressure 0.001 Pa. *Piezoelectric force microscopy (PFM*) was employed to investigate the local electromechanical (piezoelectric) properties of the polysiloxane–polyimide films using a multimode AFM setup (NT-MDT SolvePro). Essentially, PFM technique is based on the detection of the local electromechanical vibration of the sample caused by an external alternating current (AC) voltage. The voltage is applied to a conductive (platinum covered) AFM probing tip, which is used as a movable top electrode. The external driving voltage with frequency υ generates a sample surface vibration with the same frequency due to the converse piezoelectric effect. The modulated deflection signal from the cantilever, which oscillates together with the sample, is detected using the lock-in technique. The amplitude of the first harmonic signal from the lock-in amplifier is a function of the magnitude of piezoelectric displacement and phase shift between the AC electric field and the cantilever displacement. Thus, during AFM scanning of the sample surface, the magnitude of the AFM tip displacement is recorded simultaneously with the phase of the displacement (orientation of the piezoelectric response). This means that regions with opposite piezoelectric orientation will vibrate in the counter phase with respect to each other under the applied electric field [55]. The amplitude of displacement allows us to estimate the magnitude of the piezoelectric response, while the phase image provides information on the sequence of the mechanical oscillation that is related to the orientation of the polarization, allowing the identification of domain structures. In this study, all images were taken during one imaging session with the same cantilever (nominal spring constant 0.1 N m^−1^ and free resonant frequency of 245.9 kHz) and laser position in order to allow for quantitative comparison across the samples investigated. PFM images (surface topography, magnitude, and phase) were obtained in contact mode at a vertical deflection setpoint of 2 nm, drive voltage of 1 V, scan rate of 0.5 Hz, and surface area of 20 × 20 mm^2^.

### 3.3. Procedures

#### 3.3.1. Synthesis of Poly(siloxane-imide) Copolymers PI1, PI2, and PI5

The statistical copolymers polyimide–polydimethylsiloxane with nitrile functional groups (Scheme 1) were prepared following a previously described procedure [26,27]. Both 4,4′-Oxydiphthalic anhydride (ODPA) and other reagents were provided by Aldrich and used as received; 2,6-Bis(3-aminophenoxy)benzonitrile (DA-CN) was synthesized by a nucleophilic displacement reaction of 2,6-dichlorobenzonitrile and 3-aminophenol, following a method previously reported [26]. DA-Silox1 and DA-Silox2 were synthesized by equilibration of the cyclic siloxane tetramer, [(CH_3_)_2_SiO]_4_ (octamethylcyclotetrasiloxane), with 1,3-bis(3-aminopropyl)tetramethyldisiloxane in a pre-established ratio to obtain the desired molecular weight [27]. Then, PI1, PI2, and PI5 were obtained in two steps. In the first step, solutions of PAA-Silox1,2,5 were prepared from polycondensation reactions of ODPA with mixtures of two diamines, DA-CN and DA-Silox1,2,5. In the second step, PAA-silox1,2,5 were converted to the corresponding imide structure PI1,2,5 using chemical imidization.

The poly(amic acid)s were synthesized at room temperature, under nitrogen atmosphere. ODPA (0.310 g, 1 mmol), N-methy-2-pyrrolidone (NMP) (2 mL), and tetrahydrofuran (0.5 mL) were introduced into a three necked flask. An amine-terminated oligodimethylsiloxane DA-Silox1 (0.109 g, 0.108 mmol) solution in THF (0.5 mL) was added and the reaction was continued under stirring for 1.5h. Then, a solution of DA-CN (0.2825 g, 0.891 mmol) in NMP (1.5 mL) was added and the stirring was continued for 10 h. A yellow viscous solution of PAA-Silox1 was thus obtained. The imide structure PI1 was performed by chemical imidization of PAA-Silox1 by adding acetic anhydride (2 mL) and pyridine (1 mL) to the viscous solution of PAA-Silox1. The THF was distilled off under vacuum at 50 °C and then the reaction mixture was heated under stirring at 130 °C for 4 h. After cooling to room temperature the solution was precipitated in a large quantity of water. The resulting precipitate was filtered, washed again with water, and dried at 80 °C for 4 h and at 110 °C for 5 h (Yield = 87%).

PI2 and PI5 were prepared following the same procedure, by using ODPA (0.310 g, 1 mmol), DA-Silox2 (0.140 g, 0.098 mmol), DA-CN (0.286 g, 0.902 mmol) for PI2, and ODPA (0.310 g, 1 mmol), DA-Silox5 (0.112 g, 0.45 mmol), DA-CN (0.174 g, 0.55 mmol) for PI5.

The structures (Scheme 1) were checked using FTIR spectroscopy (Figure S1) and ^1^H-NMR (Figure S2) spectroscopy and GPC analysis. The molecular weight of the oligomers (α,ω-bis(aminopropyl)oligodimethylsiloxane) was as follows: for PI1 it was Mn = 1280 g mol^−1^, for PI2 Mn = 1430 g mol^−1^, and for PI5 Mn = 248 g mol^−1^. The molecular weight of the final polyimide–polydimethylsiloxane copolymers determined with GPC measurements was Mn,***_PI1_*** = 21,200 g mol^−1^, Mn,***_PI2_*** = 12,015 g mol^−1^, and Mn,***_PI5_*** = 20,090 g mol^−1^. The specific IR absorption bands (KBr, cm^−1^): PI1: 744 m (C=O bending), 798 s (C–H from Si–CH_3_ stretching), 1099–1026 s (Si–O–Si stretching), 1261 s (C–H deformation in Si–CH_3_), 1371 s (C–N stretching of imide), 1605 m (C=O from CONH stretching vibration), 1722 vs. (C=O symmetric stretching), 1780 m (C=O asymmetric stretching), 2232 w (C≡N from the nitrile group), 2962 m (C–H from Si−CH_3_ asymmetric stretching), 3200–3700 m (OH and NH from COOH and CONH stretching vibration), PI2: 743 m (C=O bending), 797 s (C–H from Si–CH_3_ stretching), 1099–1026 s (Si–O–Si stretching), 1261 vs. (C–H deformation in Si–CH_3_), 1371 s (C–N stretching of imide), 1571 s (C–NH), 1724 s (C=O symmetric stretching), 1781 s (C=O asymmetric stretching), 2232 m (C≡N from the nitrile group), 2962 s (C−H from Si–CH_3_ asymmetric stretching), 3000–3500m (OH and NH from COOH and CONH stretching vibration), PI5: 744m (C=O bending), 780m (C–H from Si–CH_3_ stretching), 1098–1026 s (Si–O–Si stretching), 1274 s (C–H deformation in Si–CH_3_), 1368 s (C–N stretching of imide), 1715 vs. (C=O symmetric stretching), 1778 m (C=O asymmetric stretching), 2232 w (C≡N from the nitrile group), 2952 m (C–H from Si–CH_3_ asymmetric stretching), 3000–3600 m (OH and NH from COOH and CONH stretching vibration). ^1^H-NMR (CDCl_3_-d^6^, ppm) of PI1 and PI2: 6.8–8.0 (aromatic protons), 3.5 (–C***H***_2_–CH_2_–CH_2_–Si–), 1.6 (–CH_2_–C***H***_2_–CH_2_–Si–), 0.5 (–CH_2_–CH_2_–C***H***_2_–Si–), 0 (C*H_3_*–Si); PI5: 6.6–8.2 (aromatic protons), 3.54 (–C***H***_2_–CH_2_–CH_2_–Si–), 1.6 (–CH_2_–C***H***_2_–CH_2_–Si–), 0.5 (–CH_2_–CH_2_–C***H***_2_–Si–), 0.0 (C***H***_3_–Si).

#### 3.3.2. Preparation of Elastomeric Composites

A pre-established amount of grounded polyimide-polydimethylsiloxane statistical copolymer was added to the PDMS solution in chloroform, followed by stirring, in order to obtain a homogenous mixture (Table 1). In the next step, the crosslinker agent (TEOS) and catalyst for condensation and crosslinking (DBTDL) were added to each reaction mixture, continuing the stirring for 1 h at room temperature. The solution mixture was poured into Teflon molds in the fume hood and left for crosslinking at room temperature for 24 h. For each sample preparation, 9 mL CHCl_3_ were used as solvent for the PDMS matrix and 0.01 g DBTDL as a catalyst. The films were removed from the Teflon molds and left standing for aging in the atmosphere on the lab bench for ten days before testing. Films were named PIa-b%, where a is the type of statistical copolymers polyimide–polydimethylsiloxane (PI1, PI2, PI5), and b the wt% PIa relative to siloxane matrix—in this paper 0, 2, 5, 10, and 20 wt% relative to siloxane weight were used (Table 1).

#### 3.3.3. Preparation of Electroactive Piezoelectric Surfaces and Humidity Sensors

A novel laboratory-scale single step process for producing low-cost, scalable film-shaped sensors with piezoelectric properties without poling and with potential application as pressure sensors in smart surfaces and “smart skin” was developed. The stretchable films (<100 µm thick) with a surface area of 50 × 50 mm were covered with a thin layer (<20 nm thick) of conductive metal (Al) using PLD. Due to the surface metal, there is no stray capacitance between the metal layer as electrode and the polymer as dielectric, which could hamper the measurements for sensor capabilities. The pressure sensors are ready for immediate use after the metal layer deposition. The tests were performed in atmospheric conditions (25 °C and 60% RH). The pressure was simulated by the fall of a steel ball with a mass of 30 g from a height of 10 cm (Figure 8) [56]. The resulting recorded voltage spike is a function of the piezoelectric properties of each film tested.

## 4. Conclusions

New composite materials based on PDMS incorporating different percentages of poly(imide-co-siloxaneimide) copolymers with different segment ratios have been developed. They have been shown to have pressure-sensing and energy-harvesting properties for self-powered applications. The devices in the form of thin films show a combination of properties specific to the two classes of polymers: flexibility and elasticity specific to siloxanes and piezoelectric behavior specific to polyimides. The electrical currents generating capabilities of the composites are similar to those of traditional piezorezistive materials at *d*_33_~2.5 pm/V, with a limit of detection of 0.005 N and a maximum voltage of 750 mV harvested. Also, the dielectric properties of the samples allow such materials to function as electrostatic actuators with maximum lateral strains of ~4.7% for samples with polyimide copolymers PI1 and PI2, and 6.12% for samples with polyimide copolymer PI5. The thin films of piezoelectric composites are intrinsically ready for scalable production and integration with any irregular surfaces for simultaneous pressure-sensing and energy-harvesting from any type of kinetic and/or pressure action. Therefore, the materials presented in this paper could enable lightweight portable, wearable devices, working as both sensing and energy source materials, although these applications need a further step of optimization of the piezoelectric properties of the materials.

## Figures and Tables

**Table 1:** Composition of the samples tested.

Sample	wt% PIa Relative to Siloxane	PDMS, g	PIa, g	TEOS, g
**PDMS**	0	0.5	0	0.035
**PIa-2%**	2	0.5	0.0125	0.035
**PIa-5%**	5	0.5	0.025	0.035
**PIa-10%**	10	0.5	0.05	0.035
**PIa-20%**	20	0.5	0.1	0.035

**Table 2:** Water and ethylene glycol contact angle values for composite films.

Sample		Contact Angle (Degrees)	
		Water	Ethylene Glycol
**PDMS**		115 ± 0.37	103 ± 0.53
**PI ^a^**		93 ± 1.33	69 ± 2.67
**PI1**		98 ± 1.47	98 ± 2.01
**PI2**		105 ± 3.32	88 ± 0.71
**PI5**		91 ± 1.72	71 ± 1.33
**PI1**	**2%** **5%** **10%** **20%**	113 ± 1.52 112 ± 0.59 117 ± 0.30 127 ± 4.07	107 ± 0.76 106 ± 1.55 110 ± 0.97 127 ± 2.12
**PI2**	**2%** **5%** **10%** **20%**	115 ± 1.84 112 ± 2.23 124 ± 3.43 127 ± 3.19	107 ± 0.58 105 ± 2.98 113 ± 1.32 125 ± 3.82
**PI5**	**2%** **5%** **10%** **20%**	116 ± 0.28 114 ± 1.17 114 ± 1.13 116 ± 0.57	110 ± 0.28 109 ± 0.67 109 ± 0.55 109 ± 1.24

^a^ Full organic polyimide (without siloxane diamine, x = z = 1, y = 0 in Scheme 1).

**Table 3:** Mechanical properties of the series of samples with PI1, PI2, and PI5.

Sample	Sm,% ^a^	Y, MPa ^b^	T_nm_, MPa ^c^	UTT, J/m^3 d^
**PDMS**	1015	0.23	0.14	9.6
**PI1-2%**	1075	0.23	0.3	16.3
**PI1-5%**	1165	0.54	0.27	18.3
**PI1-10%**	500	0.88	0.3	12.1
**PI1-20%**	340	2.24	0.48	13.5
**PI2-2%**	960	0.22	0.29	14.4
**PI2-5%**	1053	0.3	0.28	16.9
**PI2-10%**	400	1.4	0.38	12.1
**PI2-20%**	220	6.6	0.88	17.4
**PI5-2%**	800	0.19	0.15	7.1
**PI5-5%**	1032	0.2	0.27	12.5
**PI5-10%**	623	0.2	0.2	7.8
**PI5-20%**	425	0.3	0.17	4.9

^a^ elongation at break; ^b^ Young’s modulus (calculated at 5% elongation); ^c^ stress at break; ^d^ tensile toughness (calculated as the area from the stress—strain curve).

**Table 4:** Roughness and piezoelectric coefficient for the samples tested.

Sample	R (nm)	*d*_33_ (pm/V)
**PDMS**	0.5	0
**PI**	2.1	2.4 ± 0.5
**PI1**	6	6.1 ± 1.5
**PI1-2%**	4	0.5 ± 0.1
**PI1-5%**	59	1.0 ± 0.2
**PI1-10%**	150	1.5 ± 0.4
**PI1-20%**	157	1.7 ± 0.3
**PI2**	2.6	5.5 ± 1
**PI2-2%**	9	2.5 ± 0.5
**PI2-5%**	11	2.5 ± 0.5
**PI2-10%**	33	2.0 ± 0.5
**PI2-20%**	68	2.5 ± 0.4
**PI5**	2.2	2.0 ± 0.5
**PI5-2%**	6	0.4 ± 0.1
**PI5-5%**	8	0.5 ± 0.1
**PI5-10%**	9	1.6 ± 0.3
**PI5-20%**	12	1.5 ± 0.3

## Data Availability

The data presented in this work are available in the article and Supplementary Materials.

## Supplementary Materials

The following supporting information can be downloaded at: https://www.mdpi.com/article/10.3390/molecules27238524/s1, Figure S1. FTIR spectra for the polyimide–polydimethylsiloxane copolymers PI1, PI2, and PI5; Figure S2. ^1^H NMR spectra for the polyimide–polydimethylsiloxane copolymers PI1, PI2, and PI5; Figure S3. Cryofracture SEM images of the composite films; Figure S4. Surface energy parameters of the composite films; Figure S5. Elastoplastic behavior of the composite films under cyclic stress loads; Figure S6. Dielectric properties of the composite samples with 5 wt% polyimide compared with reference samples in normal conditions at room temperature (a), atmosphere saturated with water vapors (b), and after immersion in distilled water (c) [57,58,59,60,61].

## Sample Availability

Samples of the compounds are not available from the authors.
