# Supplementary material for: All-Polymer Piezo-Composites for Scalable Energy Harvesting and Sensing Devices
Source: Molecules. 2022 Dec 3;27(23):8524. doi: 10.3390/molecules27238524 (PMC9741343; doi:10.3390/molecules27238524)
Supplement: Supplementary file 1 [file molecules-27-08524-s001.zip › molecules-2005853-supplementary.pdf]

All-Polymer Piezo-Composites for Scalable Energy Harvesting and Sensing Devices

George-Theodor Stiubianu,[1] Adrian Bele,[1] Alexandra Bargan,[1] Violeta Otilia Potolinca,[2] Mihai Asandulesa,[3] Codrin Tugui,[1] Vasile Tiron,[4] Corneliu Hamciuc,[5] Mihaela Dascalu,[1*] Maria Cazacu[1]

[1] Department of Inorganic Polymers,"Petru Poni" Institute of Macromolecular Chemistry, Aleea Grigore Ghica Voda 41A, 700487 Iasi, Romania

[2] Department of Polyaddition and Photochemistry,"Petru Poni" Institute of Macromolecular Chemistry, Aleea Grigore Ghica Voda 41A, 700487 Iasi, Romania

[3] Department of Electroactive Polymers and Plasmochemistry,"Petru Poni" Institute of Macromolecular Chemistry, Aleea Grigore Ghica Voda 41A, 700487 Iasi, Romania

[4] Research Center on Advanced Materials and Technologies, Department of Exact and Natural Sciences, Institute of Interdisciplinary Research, Alexandru Ioan Cuza University of Iasi, Bd. Carol I nr. 11, Iasi, 700506, Romania

[5] Polycondensation and Thermostable Polymers,"Petru Poni" Institute of Macromolecular Chemistry, Aleea Grigore Ghica Voda 41A, 700487 Iasi, Romania

**Correspondence: amihaela@icmpp.ro, mcazacu@icmpp.ro*

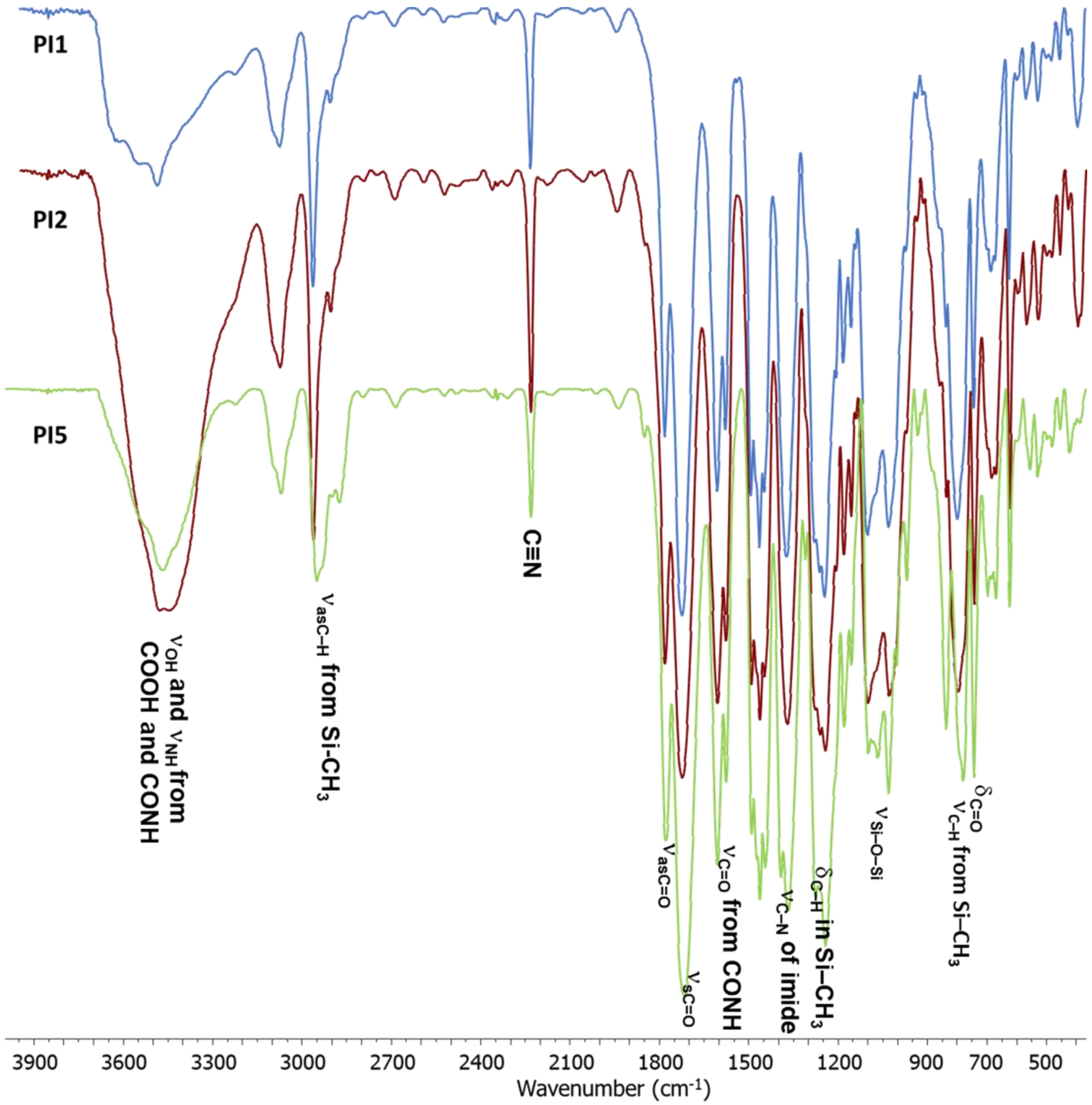

Figure S1. FTIR spectra for the polyimide-polydimethylsiloxane copolymers PI1, PI2, PI5

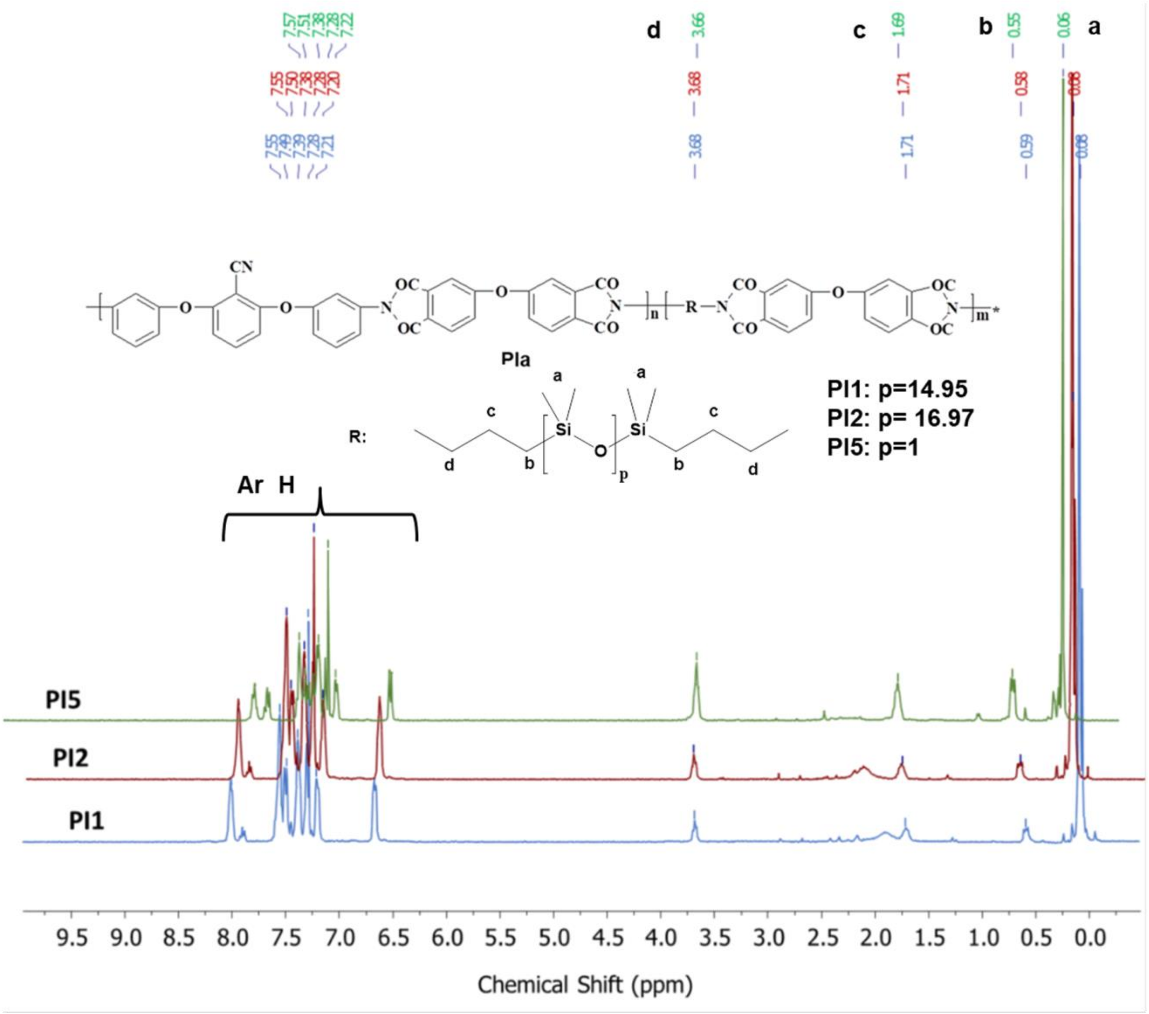

Figure S2. ^{1}H NMR spectra for the polyimide-polydimethylsiloxane copolymers PI1, PI2, PI5

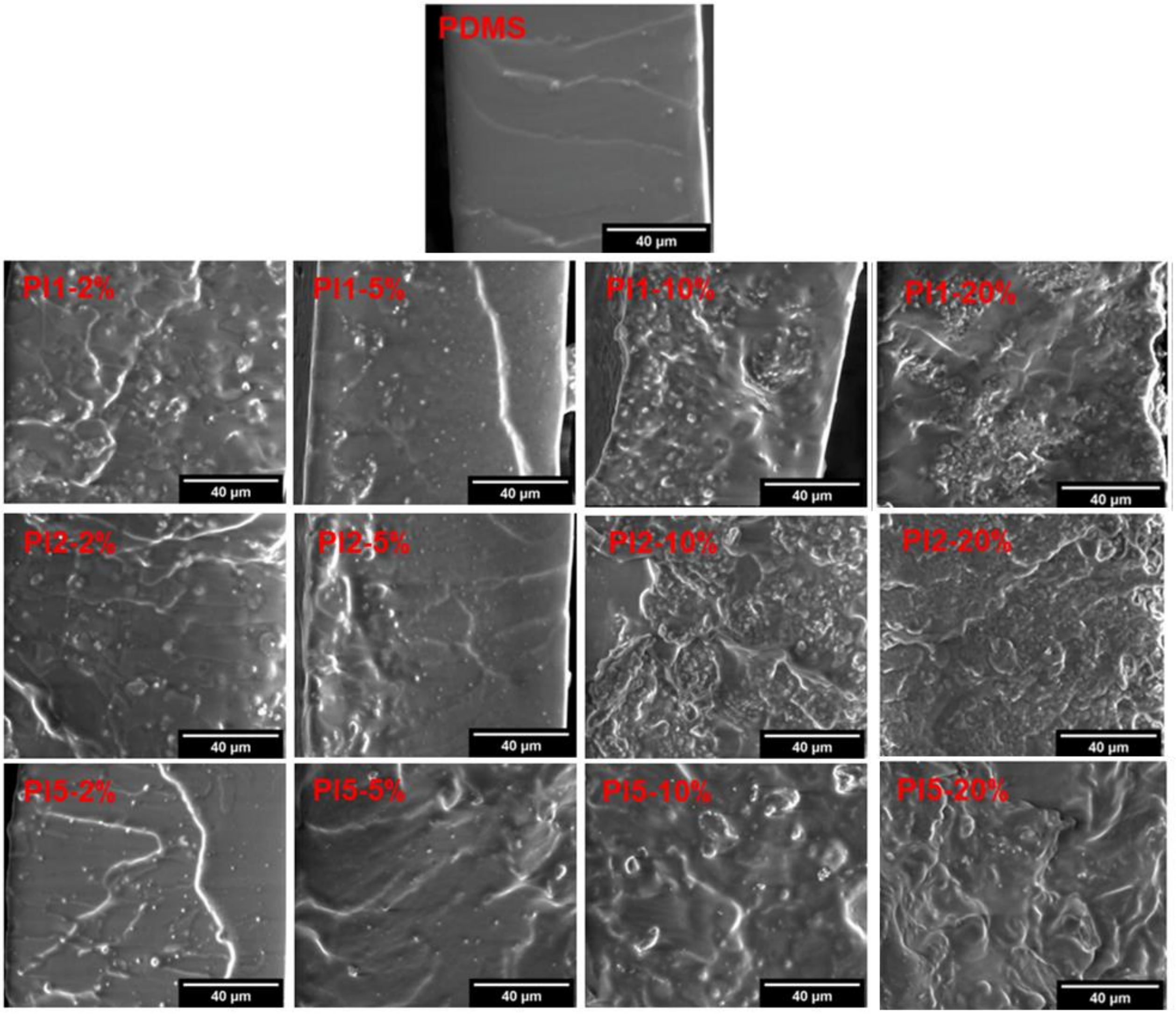

Figure S3. Cryo-fracture SEM images of the PIa-b composite films, recorded by using Large-Field Detector (LFD)

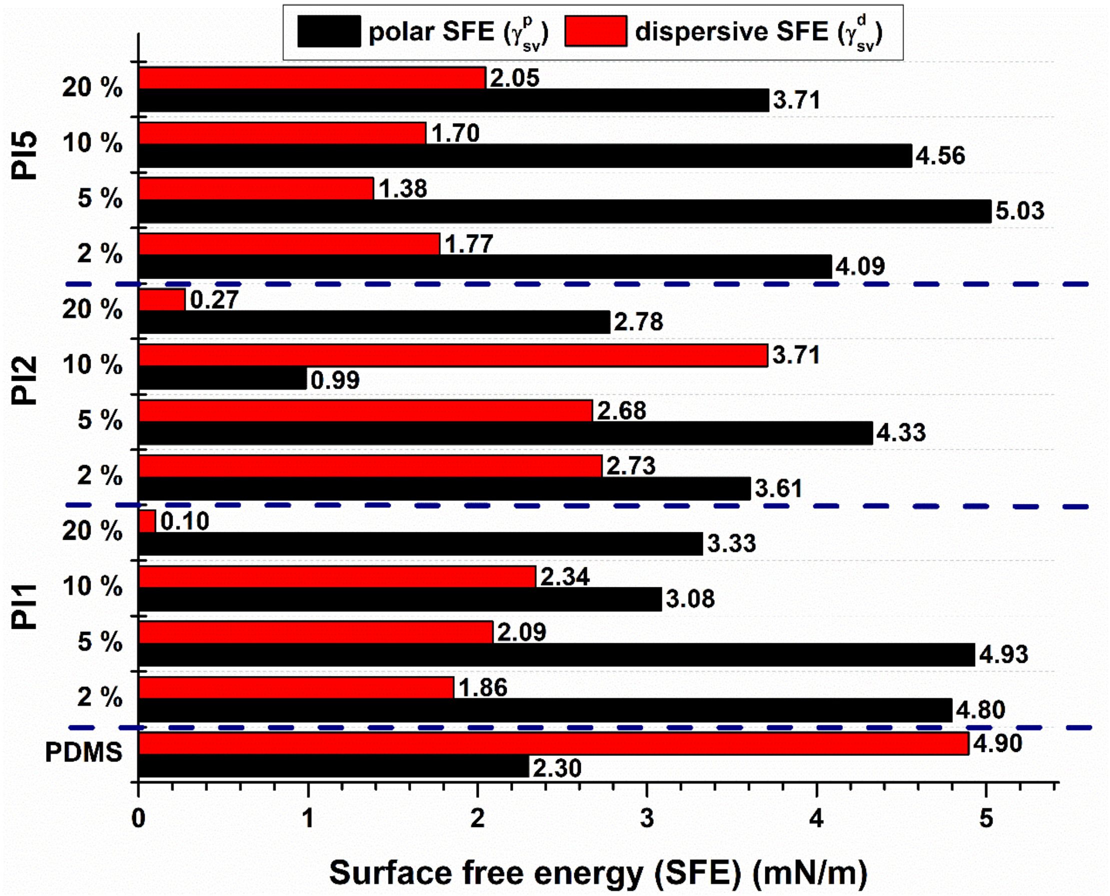

Figure S 4. Surface energy parameters of the composite films calculated by using two references liquids (water and ethylene glycol).

Ethylene glycol is among the standard test liquids used for the determination of the surface free energy and is commonly used for this purpose [1-5]. Ethylene glycol has a surface tension similar to that of diiodomethane, being less toxic. Water has the polar component higher than the dispersive one, while ethylene glycol has the dispersive component higher than the polar one. Thus, we have used these two liquids to calculate the surface free energy.

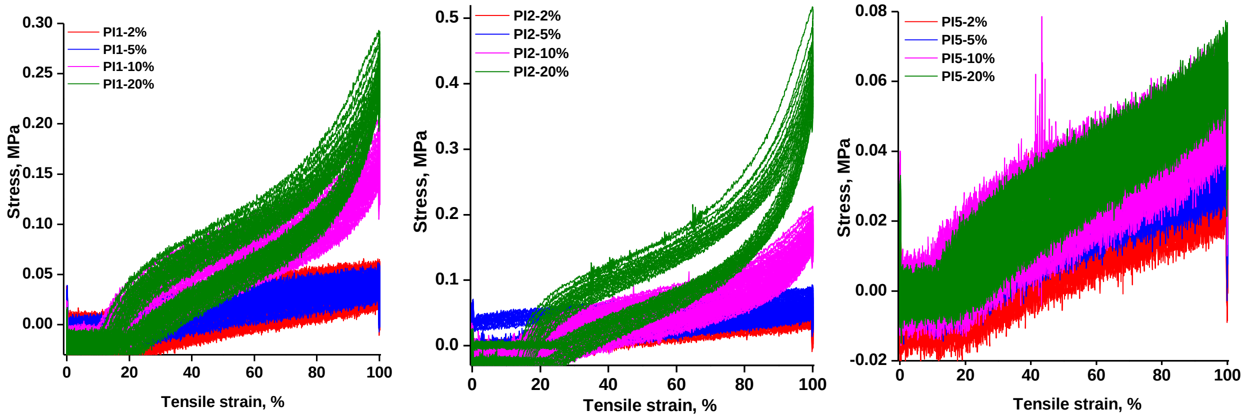

Figure S 5. Elastoplastic behavior of the composite films under cyclic stress loads

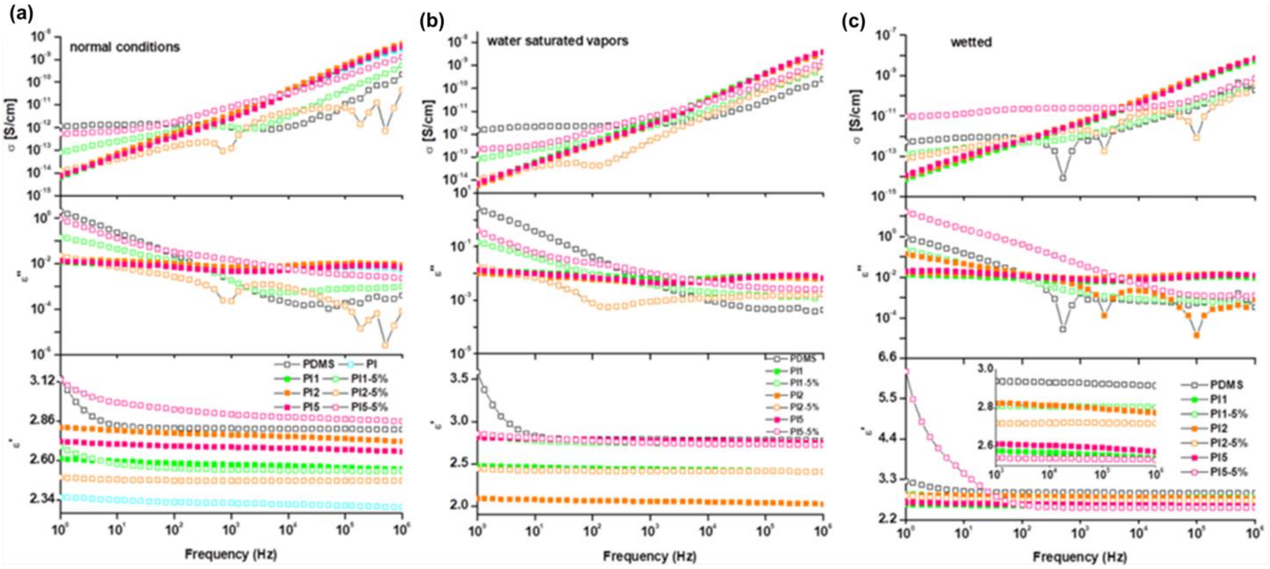

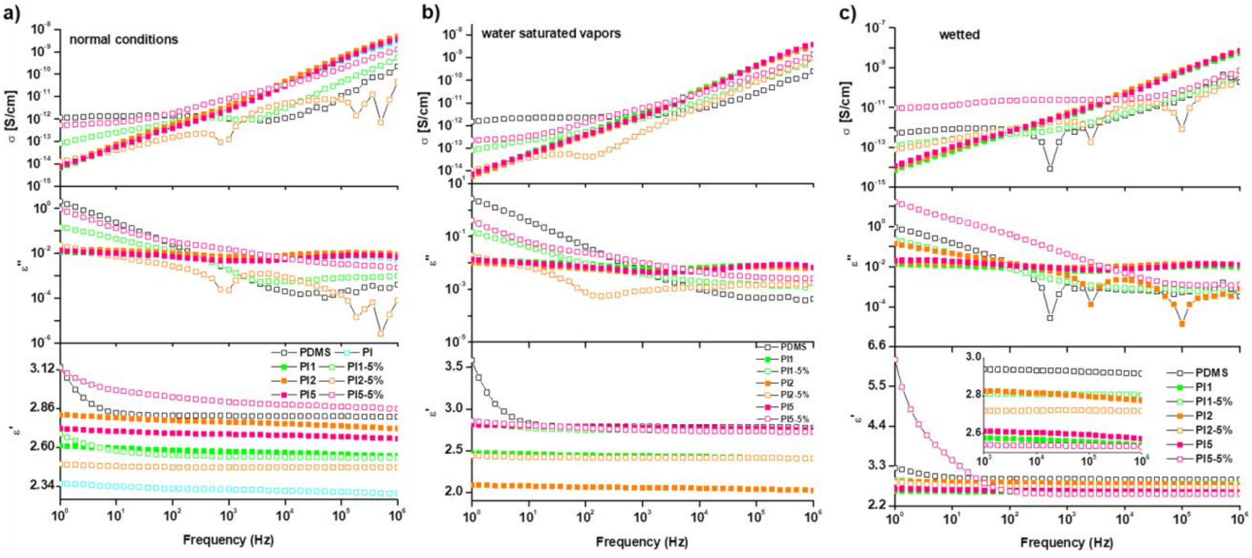

Figure S6. Dielectric properties of the composite samples with 5 wt% polyimide compared with reference samples in: normal conditions at room temperature (*a*), atmosphere saturated with water vapors (*b*), and after immersion in distilled water (c).

References

1. Pulyalina, A.; Tian, N.; Senchukova, A.; Faykov, I.; Ryabikova, M.; Novikov, A.; Saprykina, N.; Polotskaya, G. Application of cyclized polyacrylonitrile for ultrafiltration membrane fouling mitigation, *Membranes* **2022**, *12(5)*, 489.
2. Park, J.; Kang, Y.-C. Effect of radio frequency power on the physicochemical properties of MoS2 films obtained by rf magnetron sputtering, *Bull. Korean Chem. Soc.* **2016,** *37*, 1326-1330.
3. Choi, S.; Kang, J.; Park, J.; Kang, Y.-C. Tin nitride thin films fabricated by reactive radio frequency magnetron sputtering at various nitrogen gas ratios, *Thin Solid Films*, **2014**, *571*, 84-89.
4. Zheng, G.; Zhang, D.; Zheng, C.; Yao, Y.; Long, Z. Facile fabrication of biomimetic superoleophobic composite coating via Schiff base reaction and self-assembly, *Progress in Organic Coatings*, **2020,** *142*, 105568.
5. Yoshioka, T.; Fujita, H.; Kimura, Y.; Hattori, Y.; Kitamura, M. Wide-range work function tuning in gold surfaces modified with fluorobenzenethiols toward application to organic thin-film transistors, *Flexible and Printed Electronics,* **2020,** *5(1)*, 014011.
